# From positive psychology to practical empowerment: theoretical construction and pathway innovation in graduate student mental health education

**DOI:** 10.3389/fpsyg.2026.1821535

**Published:** 2026-06-11

**Authors:** Lei Sun, Die Hu, Mei Su, Xuekun Xing

**Affiliations:** 1 School of Public Health, Guilin Medical University, Guilin, China; 2 Guangxi Key Laboratory of Environmental Exposomics and Entire Lifecycle Health, School of Public Health, Guilin Medical University, Guilin, China

**Keywords:** educational intervention, graduate student mental health, positive psychology, practical pathways, psychological capital, theoretical construction

## Abstract

The graduate student population faces unique pressures from academics, employment, interpersonal relationships, and various other stressors, with their mental health issues increasingly drawing attention. Traditional mental health education models often focus on problem correction and crisis intervention, whereas the perspective of positive psychology emphasizes discovering and cultivating individuals’ strengths, resilience, and wellbeing; it offers a new theoretical paradigm for graduate student mental health education. This paper aims to systematically review the application of core theories of positive psychology in the field of graduate student mental health education and, based on existing empirical research, thoroughly explore effective pathways for translating theoretical construction into specific practice. The article analyzes the integration of positive psychological qualities cultivation into the entire process of graduate education and evaluates the effectiveness and challenges of different intervention models, with the goal of providing theoretical foundations and practical references for constructing a systematic, developmental, and preventive mental health support system for graduate students.

## 1 Introduction

Postgraduate education, as a critical stage for cultivating high-level innovative talents, is of paramount importance. However, these students face severe mental health challenges. Factors such as high-intensity research pressure, uncertain career prospects, complex interpersonal relationships (especially student-teacher relationships), and economic burden collectively create a high-risk environment for psychological issues among postgraduate students (Gallea et al., 2021). Research indicates that graduate students face significant mental health challenges, with a considerable proportion experiencing anxiety, depression, or high occupational burnout (Guthrie et al., 2018). For instance, a survey of university faculty and graduate students found that approximately 20% of staff and 30% of graduate students screened positive for potential depression or anxiety (Carr et al., 2022). Another survey targeting Chinese graduate students showed that 33.9% of respondents had depressive symptoms, and 20.9% had anxiety symptoms (Liang et al., 2021). Occupational burnout is also prevalent within this demographic; for instance, 8.6% of graduate students specializing in oral pathology and oral medicine in Brazil reported experiencing burnout (Maia-Lima et al., 2026). Furthermore, their levels of psychological distress may be higher than those of other populations. An international study found that 13.4% of mental health researchers scored above the cutoff for severe psychological distress, with a significantly higher proportion among graduate-level participants (Hill et al., 2022). Other studies corroborate the severity of the issue; for example, a survey found that 40.8% of graduate students were identified as being at current risk for suicide (Abreu et al., 2021). Most graduate students experienced heightened levels of loneliness and depressive symptoms (Dip et al., 2026a), along with significantly reduced social interactions and less frequent communication with faculty (Dip et al., 2026b). Furthermore, online teaching lacked an immersive quality, resulting in suboptimal learning experiences for students (Layman-Lemphane et al., 2026), thereby exacerbating their psychological burden. For international students, pandemic-related stress is significantly associated with anxiety and depression, and educational level (undergraduate versus postgraduate) plays a moderating role in this relationship (Reid et al., 2026). Traditional mental health services often focus on the diagnosis and treatment of existing problems, belonging to a “deficit model.” In contrast, positive psychology shifts the research focus to human strengths, virtues, and the conditions for achieving a fulfilling life, advocating a “health model.” Introducing positive psychology into postgraduate mental health education means shifting from a passive response to active cultivation, aiming to enhance postgraduate students’ psychological resilience, subjective wellbeing, sense of meaning, and academic self-efficacy, thereby strengthening their internal resources for coping with adversity.

Empirical research provides strong support for this shift in mental health education approaches. For example, studies have found that graduate students’ academic career adaptability partially mediates the relationship between daily stress and mental health, indicating that cultivating internal resources such as adaptability can buffer the negative impact of stress on mental health (Li et al., 2023). Furthermore, the quality of the supervisor-student relationship (SSR) significantly affects graduate students’ anxiety levels. Satisfaction with SSR is negatively correlated with anxiety, with research self-efficacy mediating this relationship and mindset (growth vs. fixed) moderating it (Li et al., 2025). This suggests that improving teacher-student interactions through positive psychology interventions and enhancing students’ sense of self-efficacy and growth mindset is an effective way to promote mental health. A literature review in the veterinary field also points out that while focusing on preventing mental illness, it is necessary to balance the promotion of mental health among veterinary students and graduates, utilizing positive psychology to cultivate and understand professional wellbeing (Bookbinder, 2025). This not only helps prevent mental illness but also promotes the comprehensive growth and excellent development of graduate students.

Therefore, it is imperative to develop a mental health education framework based on positive psychology theory, focusing on strength cultivation and resource empowerment. Such a framework should go beyond the traditional deficit-repair model and be committed to systematically developing the psychological capital of the graduate student population, such as resilience, hope, optimism, and self-efficacy (Saleem et al., 2022). Through various pathways such as curriculum intervention, behavior change programs, mindfulness training, and building supportive teacher-student relationships and peer networks, we can proactively empower graduate students. This approach helps them not only achieve academic success but also gain rich inner experiences and sustainable career development, thereby providing systematic guidance for transforming and upgrading mental health education for graduate students in China.

## 2 The Core theoretical framework of positive psychology and its implications for graduate student mental health education

### 2.1 Psychological capital theory: Hope, efficacy, resilience, and optimism

Psychological capital theory conceptualizes an individual’s positive psychological state as a developable core resource, comprising hope, self-efficacy, resilience, and optimism (Geremias et al., 2022). For graduate students in high-pressure academic environments, these constructs exhibit significant plasticity and can be enhanced through cognitive behavioral training, cultivation of hope pathways, resilience training, and other methods, and the effects of such training have demonstrated long-term stability. A sense of hope enables graduate students to set and persist in pursuing challenging academic goals and plan pathways to achieve them (Rew et al., 2024). Self-efficacy is directly related to a graduate student’s confidence level when facing complex research problems, with high self-efficacy promoting a more proactive approach to challenges (Wu et al., 2025). Psychological resilience, defined as the ability to recover and grow from adversity, is crucial for coping with common academic setbacks such as repeated experimental failures and manuscript rejections (Yang et al., 2023). Optimism, as a positive explanatory style, helps graduate students reappraise academic stress as an opportunity for growth rather than a threat, thereby maintaining a positive psychological state (Wu et al., 2025). A substantial body of empirical research confirms the direct protective effect of psychological capital on mental health. For example, research among Malaysian university students found that self-efficacy and optimism are significant negative predictors of psychological distress (Wu et al., 2025). Another intervention study targeting Chinese adolescents also indicated that a sports prescription based on positive rumination can effectively enhance their levels of optimism, hope, self-efficacy, and resilience, thereby improving mental health (Min and Yao, 2022). More importantly, the mechanism of psychological capital’s effect is not limited to direct influence. Research shows that psychological capital can indirectly promote mental health by enhancing an individual’s perceived level of social support (Khan et al., 2024). A study focusing on Chinese university students found that perceived social support plays a significant mediating role in the relationship between psychological capital and mental health (Khan et al., 2024). This suggests that in graduate student mental health education, interventions should go beyond single psychological skill training. They should systematically integrate the cultivation of the four qualities—hope, efficacy, resilience, and optimism—into the entire educational process and focus on building supportive social networks. This approach aims to comprehensively enhance graduate students’ psychological capital and mental health through both direct and indirect pathways.

### 2.2 PERMA wellbeing model and self-determination theory

The PERMA model offers a multidimensional framework for understanding and enhancing the subjective wellbeing of graduate students. This model posits that wellbeing stems from five elements: Positive Emotions, Engagement, Relationships, Meaning, and Accomplishment (Jin et al., 2022). In the context of graduate education, positive emotions can be cultivated by creating a supportive academic atmosphere and recognizing incremental achievements (Shaban et al., 2025). Engagement, particularly the deeply absorbed state of “flow,” is closely related to focus and immersion in work or study, which is crucial for improving research efficiency and wellbeing (Bai et al., 2025). The Relationships element emphasizes the importance of building supportive networks, such as peer assistance and positive student–advisor relationships, which not only provide emotional support but also form the foundation for academic collaboration (Rew et al., 2024). A sense of Meaning helps graduate students connect their daily research work to broader personal values or societal contributions, thereby enhancing intrinsic motivation (Jin et al., 2022). Accomplishment involves the recognition of academic milestones, which can effectively satisfy graduate students’ need for competence (Shaban et al., 2025). Self-Determination Theory further explains the intrinsic motivational mechanisms of psychological health from the perspective of basic psychological needs. It emphasizes that the satisfaction of the three needs—autonomy, competence, and relatedness—is fundamental to intrinsic motivation and psychological health (Shaban et al., 2025). For graduate students, satisfying the need for autonomy means having a certain degree of decision-making power in research direction and methodology choices. Satisfying the need for competence requires providing tiered, manageable challenges that allow their abilities to be confirmed and enhanced. Satisfying the need for relatedness relies on fostering inclusive, respectful, and mutually supportive relationships among students, advisors, and peers (Shaban et al., 2025). Empirical research supports the practical effectiveness of these theories. For example, a study on community nurses found that psychological capital (including self-efficacy, hope, optimism, etc.) was significantly positively correlated with job satisfaction, with self-efficacy, hope, and optimism being significant predictors of job satisfaction (Shaban et al., 2025). This indirectly confirms the importance of satisfying needs such as competence (through self-efficacy), meaning (through hope), and positive expectations (through optimism). Another study indicated that perceived organizational support for the use of strengths can promote work engagement by enhancing self-efficacy and optimism (Bai et al., 2025). Therefore, graduate student mental health education should strive to create an educational environment that systematically satisfies graduate students’ needs for autonomy, competence, and relatedness by granting academic autonomy, designing appropriate challenging tasks, and fostering positive interpersonal connections. This approach comprehensively enhances their wellbeing and mental health status across the five dimensions of the PERMA model.

**Figure 1:**
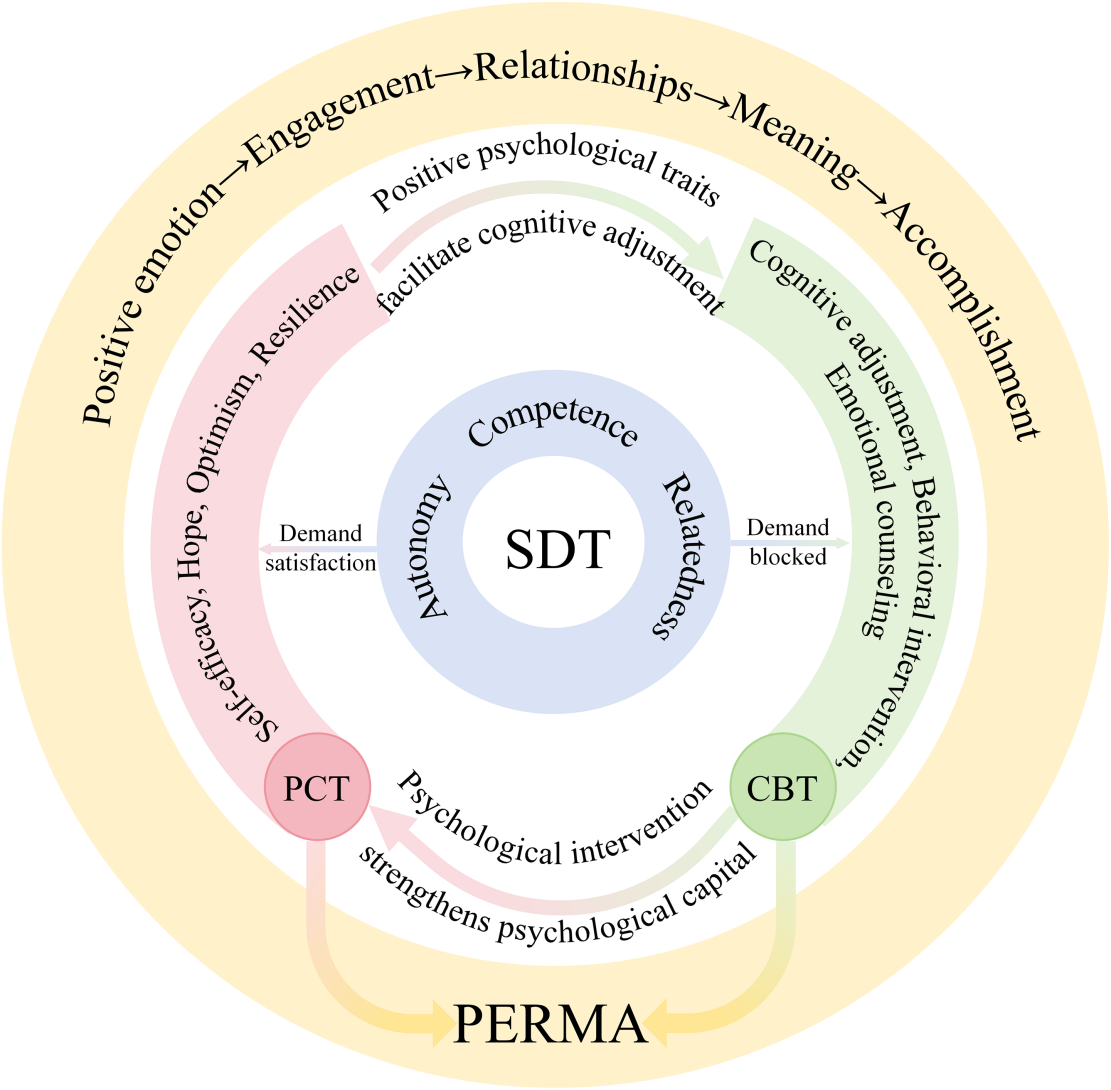
Structural diagram of multi-theory collaborative relationship. This figure intuitively presents the hierarchical logical interconnections among the PERMA wellbeing model, Self-Determination Theory, Psychological Capital Theory, and Cognitive Behavioral Therapy. Positioning Self-Determination Theory as the core intrinsic driving force, we posit that the satisfaction of its three basic psychological needs not only cultivates positive psychological capital but also necessitates Cognitive Behavioral Therapy to alleviate psychological barriers and rectify negative states; multiple elements interact synergistically, ultimately collectively influencing the five wellbeing dimensions. These theories are mutually interconnected and synergistically reinforcing, collectively illustrating the intrinsic pathway for enhancing graduate students’ mental health and subjective wellbeing.

The PERMA model, self-determination theory, psychological capital theory, and cognitive-behavioral therapy collectively form the theoretical framework for enhancing graduate students’ mental health. These four theoretical frameworks differ in their conceptual underpinnings and mechanisms yet are interdependent. Table 1 outlines the core elements, psychological logic, and specific applications within the graduate student context for each theory. These theories interact dynamically, complement and regulate one another, and ultimately collectively contribute to enhancing graduate students’ subjective wellbeing. The mechanism underlying their synergistic effects is detailed in Figure 1.

**Table 1:** Summary of internal correlation among theories.

Core theory	Constituent elements	Underlying psychological logic	Application in postgraduate scenarios
PERMA wellbeing model	Positive emotion, engagement, relationships, meaning, accomplishment	Evaluate subjective wellbeing from experiential dimensions	Comprehensively improve postgraduates’ scientific research wellbeing and mental status
Self-determination theory	Autonomy, competence, relatedness	Fundamental source generating individual intrinsic motivation	Build underlying mental support and activate intrinsic research motivation
Psychological capital theory	Self-efficacy, hope, optimism, resilience	Stable positive psychological traits	Enhance stress resistance and boost learning and work engagement
Cognitive behavioral therapy	Cognitive adjustment, behavioral intervention, emotional counseling	Correct negative thinking and behavioral patterns	Alleviate academic anxiety and interpersonal conflicts

## 3 Current status, challenges, and the necessity of positive psychology intervention for postgraduate students’ psychological health

### 3.1 Unique stressors and manifestations of psychological distress in the postgraduate student population

The stress faced by the postgraduate student population exhibits significant multiple dimensions and persistence, with its stressors primarily spanning multiple areas such as scientific research, employment, interpersonal relationships, and economics. Research pressure is one of the core stressors, characterized by the coexistence of high intensity and high uncertainty. A cross-sectional comparative study involving Canadian undergraduate and postgraduate students found that although undergraduate students generally perceived higher levels of stress in areas such as academics and learning environment, the postgraduate student population also experiences specific pressures related to academic research (Mackinnon et al., 2025). This pressure is particularly prominent among medical postgraduate students. A survey of medical postgraduate students in southeastern China showed that academic pressure is one of their main sources of distress, with second-year master’s students experiencing the highest academic pressure (Tan et al., 2022). Employment pressure is another critical dimension, which has become especially severe under the impact of external crisis events such as the COVID-19 pandemic. Research confirms that the high unemployment rate among Chinese university graduates during the pandemic led to significant mental health issues such as anxiety and depression (Peng et al., 2023). For medical postgraduate students, employment pressure is also a major psychological stressor, with third-year master’s students experiencing the highest levels of employment pressure (Tan et al., 2022). Furthermore, the quality of interpersonal relationships with supervisors has a significant impact on the psychological wellbeing of postgraduate students. An international study involving mental health researchers found an association between the work-related psychosocial stressor “interpersonal relationships and leadership” and psychological distress. Although this association weakened after controlling for mental health covariates, it still suggests that poor interpersonal relationships may constitute a stressor (Hill et al., 2022). Economic pressure and the sense of disparity arising from comparisons with peers are also factors that should not be overlooked. For example, among Chinese nursing postgraduate students, monthly income is one of the risk factors predicting their mental health status (Guo et al., 2021).

Empirical studies consistently indicate that the detection rates of anxiety, depression, and lethargy among graduate students are high. A survey among Chinese nursing graduate students found that the proportion of abnormal total scores on the Symptom Checklist-90 (SCL-90) (total score >160) reached 14.2%, with the obsessive-compulsive dimension having the highest positive detection rate at 21.5% (Li et al., 2021). Among medical post-graduate students, the detection rates of self-reported depressive symptoms and anxiety symptoms were 21.6 and 9.4%, respectively (Tan et al., 2022). External crisis events such as the COVID-19 pandemic have significantly amplified these inherent stressors, exerting additional pressure on the mental health of graduate students. Lockdowns, online teaching, social isolation, and reduced educational opportunities caused by the pandemic have collectively undermined students’ psychological resilience. A cross-sectional study targeting university students in the Kansai region of Japan showed that over 50% of undergraduate and graduate students experienced mild or higher levels of depression and anxiety, with approximately 11% experiencing severe depression and anxiety. Concerns about future prospects exacerbated the levels of depression and anxiety (Noda et al., 2021). A systematic review and meta-analysis further confirmed that the pooled prevalence rates of depression, anxiety, and stress symptoms among the student population during the pandemic were as high as 32.0, 28.0, and 31.0%, respectively (Fang et al., 2022). The isolated lifestyle brought about by the pandemic disrupted students’ daily routines, and an inverted day-night lifestyle was associated with worsening levels of depression and anxiety (Noda et al., 2021). Simultaneously, the contraction of the job market and uncertainties in graduation processes caused by the pandemic subjected graduates to unprecedented psychological challenges. A study at the University of Gondar in Ethiopia found that the prevalence rates of stress, anxiety, and depression among graduating students reached 22.2, 39.6, and 40.2%, respectively (Mekonen et al., 2021). These data collectively reveal the heavy psychological burden borne by the graduate student population due to the convergence of multiple stressors, particularly catalyzed by external crises.

### 3.2 Limitations of traditional mental health education models and the paradigm shift of positive psychology

Traditional mental health education models often follow a passive “problem-response” approach, with interventions primarily focused on individual psychological counseling and post-crisis interventions. While these services are crucial for addressing severe psychological issues that have already emerged, their coverage is often limited, and significant stigma barriers exist, deterring many graduate students in a “sub-health” state or those without a clinical diagnosis but seeking better psychological status. The limitation of this model lies in its failure to view mental health promotion as a normalized, universal component of the graduate training system. For example, a qualitative interview study targeting Chinese medical graduate students pointed out that current mental health education is often conducted by distributing mental health education manuals and holding lectures, lacking proactive, tiered interventions for students at different stress levels (Zhang et al., 2024). This “wait-and-see” service model struggles to reach the broad graduate student population affected by common issues such as research pressure, employment anxiety, and interpersonal difficulties, who do not yet meet diagnostic criteria. Furthermore, the traditional model emphasizes “repairing damage,” intervening only after students exhibit obvious symptoms, overlooking the importance of building psychological resilience and strength before problems arise.

The rise of positive psychology has brought about a paradigm shift in graduate student mental health education, moving from “repairing damage” to “building strengths.” This view advocates the preemptive, universal, and curricular integration of mental health education, viewing it as an indispensable part of graduate students’ core competencies and comprehensive development goals. This paradigm emphasizes cultivating individuals’ internal resources, such as positive emotions, psychological capital, and resilience, to enhance their capacity to cope with stress and challenges. Research indicates that positive emotions significantly impact graduate students’ psychological capital and academic engagement behaviors (Saleem et al., 2022). Psychological capital (including hope, self-efficacy, resilience, and optimism) and social support play important mediating roles in alleviating the relationship between work stress and anxiety (Ling and Yu, 2023). For example, among physical education students, social support and psychological resilience serve as significant negative mediators between employment pressure and employment anxiety (Wang and Zhang, 2025). Consequently, systematic educational interventions designed to enhance students’ psychological capital and foster social support systems can effectively buffer the negative effects of stress, thereby establishing a foundation for long-term stress resistance. The educational model advocated by positive psychology, such as through personal counseling, flexible academic goal setting, and resilience training, can help graduate students better cope with demanding academic tasks (Saleem et al., 2022). This approach of integrating mental health education into the daily cultivation process not only helps prevent the occurrence of psychological issues but also improves students’ overall academic performance and future career adaptability. For instance, studies show that students with greater psychological resilience experience a reduced negative impact of perceived stress and anxiety on sleep quality (Du et al., 2020). Therefore, mental health education from the perspective of positive psychology transcends traditional problem remediation, shifting toward empowering students and cultivating their internal strengths and positive qualities. This aligns closely with the goals of graduate education, which pursue innovation, resilience, and comprehensive development, providing a solid theoretical foundation and practical pathway for constructing a more effective and forward-looking mental health support system for graduate students.

## 4 Theoretical construction of graduate student mental health education from the perspective of positive psychology

### 4.1 Construction of the goal system: from symptom alleviation to strength development and wellbeing enhancement

The goal system of graduate student mental health education is undergoing a profound shift from the traditional pathological perspective to an empowerment perspective informed by positive psychology. This shift constructs a hierarchical framework encompassing primary, intermediate, and advanced goals. However, there is significant heterogeneity within the graduate student population, with students from different disciplines, academic years, and backgrounds exhibiting distinct psychological needs and sources of stress, making it difficult for standardized courses to achieve targeted support. Therefore, curriculum design must be based on a preliminary needs assessment, incorporating disciplinary characteristics and training stages to construct a categorized implementation system, and be implemented progressively according to the academic year timeline, thereby effectively mitigating the drawbacks of a “one-size-fits-all” intervention. The primary goal focuses on reducing levels of psychological distress and preventing the occurrence of severe psychological problems. Research indicates that the graduate student population faces unique stressors, such as anxiety related to personal protective equipment, schedule adjustments, and virtual learning models brought about by public health crises like the COVID-19 pandemic, which significantly increase stress levels among graduate students (Rana et al., 2020). Therefore, the core of primary intervention lies in alleviating these situational pressures through timely psychological support and education, preventing them from evolving into more serious psychological disorders. Achieving primary goals requires differentiated responses tailored to the specific characteristics of different disciplines. In the case of clinical majors such as medicine and nursing, it is essential to prioritize the integration of content related to compassion fatigue and occupational stress; for STEM majors, it is necessary to strengthen the management of experimental stress and the development of coping strategies for research setbacks. The intermediate goal aims to cultivate core positive psychological qualities in graduate students, such as psychological capital and a growth mindset, to enhance their internal resources for coping with stress. Developmental systems theory points out that the interaction between individuals and their environment shapes their developmental trajectory, and specific psychological factors play a key role within institutional contexts or channels with varying levels of influence (Goyer et al., 2021). For example, in less supportive academic environments, a stronger sense of belonging and a growth mindset better predict students’ academic progress (Goyer et al., 2021). This suggests that mental health education needs to systematically cultivate these malleable psychological resources to enhance students’ resilience in complex academic environments. Intermediate goals should be progressively aligned with the stages of the academic year: the first semester focusing on academic adaptation and strength identification, the second semester emphasizing stress management and psychological capital enhancement, and the graduation year prioritizing hope and career adaptability. The advanced goal is dedicated to promoting the comprehensive flourishing and wellbeing of graduate students, enabling them to gain a sense of meaning, accomplishment, and lasting happiness in their academic careers. This goal transcends problem-solving and points to the core of positive psychology—happiness. Positive psychological interventions based on the PERMA model have been empirically proven effective in enhancing the wellbeing of cancer patients and alleviating psychological distress such as anxiety and depression (Wang et al., 2025). This provides inspiration for graduate education: by integrating modules on strengths, positive emotions, relationships, meaning, and accomplishment, graduate students can be guided to unify personal value with social contribution, achieving a higher level of psychological flourishing. The achievement of high-level goals requires meaning construction aligned with disciplinary specificity. For example, humanities and social science students prioritize writing burnout and the enhancement of their sense of meaning, while arts and physical education students address practical pressures and time management. This evolution of goals from “treating existing illness” to “preventing future illness” and then to “promoting development” marks a fundamental innovation in the philosophy of graduate student mental health education.

### 4.2 Content system construction: integrating diverse positive psychological intervention modules

To achieve the aforementioned multi-level objectives, the content system for graduate student mental health education needs to integrate a series of evidence-based, diverse positive psychological intervention modules. The core modules of the course encompass multiple dimensions, including positive emotional experiences, resilience training, strengths identification, meaning construction, and fostering positive relationships. The content is tailored based on disciplinary differences and year levels. Research has confirmed that systematic positive psychology courses can significantly enhance students’ wellbeing, positive emotions, and mental health (Salois et al., 2025).

The cognitive restructuring module is foundational to the overall intervention framework. Based on cognitive-behavioral therapy (CBT) and positive psychology, it aims to teach graduate students to identify and challenge automatic negative thoughts, fostering an optimistic explanatory style and hopeful thinking. This module is closely aligned with resilience training, aimed at bolstering the capacity to cope with adversity. Courses for graduate students must address the prevalent challenges unique to this cohort, such as research pressure, employment anxiety, and advisor-student relational conflicts. Academic and employment pressures can affect anxiety levels through the mediating role of psychological resilience (Wang and Zhang, 2025; Yang et al., 2025), underscoring the necessity of establishing resilience training as a core compulsory module. CBT has been confirmed as an effective intervention for managing severe fatigue, part of its mechanism involves altering patients’ cognitive assessment of fatigue, i.e., a “response shift” occurs (Müller et al., 2023). In education, this means not only correcting irrational beliefs but also proactively constructing positive cognitive patterns, such as a growth mindset that views challenges as opportunities for growth. For students in STEM fields, optimistic attribution training regarding paper rejections should be prioritized; for those in the humanities and social sciences, cognitive restructuring to address writing burnout should be emphasized.

The strengths identification and application module directly serves the goal of strengths development. Through strengths assessment and the design of activities, it helps graduate students discover and apply their character strengths in research and daily life. Research indicates that when students are in a supportive institutional pathway, perseverance can better predict the achievement of goals such as on-time graduation (Goyer et al., 2021). Therefore, educational programs should consciously create opportunities for students to identify and apply their strengths in practice, thereby enhancing self-efficacy and academic engagement. The advantage module is a key mediator linking professional development and wellbeing (Sun, 2022). It should be integrated into the development of the advisor-advisee relationship in the first semester to identify strengths, thereby helping students build confidence in their academic endeavors.

The mindfulness and emotion regulation module aims to improve emotional awareness and management skills, reduce rumination, and increase positive emotional experiences by introducing techniques such as mindfulness meditation and compassion training. Aerobic exercise, as a method of mind–body regulation, has been proven effective in managing pain for patients with fibromyalgia (Manojlović and Kopše, 2023). This suggests that combining physical activity with mindfulness practice may have a synergistic effect on alleviating the physical and mental stress of graduate students and enhancing emotional resilience. This module should span the first academic year as a foundational tool for stress management, while also incorporating specialized activities such as “compassion fatigue group discussions” to address the needs for clinical emotional regulation of medical students (Sosnicki and Reynolds, 2026).

The relationship-building module focuses on constructing high-quality teacher-student relationships and peer support networks, training in communication skills, conflict resolution, and gratitude expression. In clinical medical education, effective interaction, collaborative learning, and trust relationships between teachers and students, as well as doctors and patients, have been identified as key facilitators for cultivating resilience (Jalali et al., 2025). For graduate students, establishing a supportive academic community is crucial. This not only provides emotional support but is also an important pathway for academic socialization. High-quality teacher-student relationships are significantly associated with lower levels of anxiety and may operate through enhancing general self-efficacy (Luo et al., 2026). Therefore, this module should be implemented preferably at the start of the school year to establish a foundation for positive relationships to support subsequent academic adaptation.

Finally, the meaning and purpose exploration module guides graduate students to integrate personal values with academic work and career planning, finding deeper meaning in research and life. In the field of psychotherapy, there are discussions on how therapists integrate personal moral values into their professional work, emphasizing collaborative exploration with clients during the treatment planning phase to set goals aligned with both parties’ values (Mcwhorter, 2019). Similarly, graduate education can draw on this approach. Through career counseling, values clarification exercises, and similar activities, it can help students anchor personal meaning in their academic exploration, facilitating a shift from external to internal motivation, thereby achieving lasting academic wellbeing and a sense of mission. This module should focus on cultivating hope, an optimistic explanatory style, and career adaptability training during the graduation year, helping students achieve a smooth transition from academia to the workforce. At the same time, differentiated content should be tailored according to disciplinary characteristics: physical education, arts, and interdisciplinary fields should focus on practical stressors and time management; STEM disciplines should strengthen resilience in the face of research setbacks; humanities and social sciences should emphasize enhancing a sense of meaning and coping with career uncertainty.

In terms of teaching methods, mere knowledge transmission should be completely abandoned in favor of emphasizing experiential and participatory learning to ensure deep integration of theoretical knowledge and practical skills. Common methods include psychodrama, group counseling, positive psychology journals, and behavioral experiments. For example, a “compassion fatigue group discussion” designed for clinical veterinary students successfully enhanced students’ knowledge, confidence, and preparedness for mental health challenges through guided sessions facilitated by clinical teachers (Sosnicki and Reynolds, 2026); online simulation education also provides new avenues for experiential learning, as an interprofessional, simulation-based psychopharmacology education activity effectively promoted collaborative learning and skill application (Bajjani-Gebara et al., 2022); after introducing design thinking into public health graduate courses, students optimally achieved learning outcomes by solving real-world challenges (Ingram et al., 2022). Course implementation must strictly follow the academic year schedule: a comprehensive needs assessment should be conducted every September to construct a multidimensional profile of “discipline + grade + needs”; the first semester should focus on adaptation education, positive emotions, strength identification, and mentoring relationship building; the second semester should focus on stress management, psychological capital enhancement, and resilience training; the graduation year should focus on hope, optimistic explanatory style, and career adaptability training.

## 5 Exploration of diversified practical pathways for graduate student mental health education

### 5.1 Curriculum integration and workshop models

Systematically integrating positive psychology content into the graduate curriculum system is an important practical pathway for constructing the theoretical framework of graduate students’ mental health education. For example, mandatory or elective courses such as “Graduate Student Mental Health and Stress Management” and “Positive Psychology and Scientific Research Innovation” can be offered, combining core concepts like psychological resilience, mindfulness, and gratitude with research practice. This curriculum integration approach can provide students with a systematic and coherent knowledge system. It helps them understand the scientific basis of mental health and learn to apply the principles of positive psychology to cope with academic stress, enhance innovative thinking, and maintain research motivation (Zhang et al., 2020). Research indicates that an 8-week, classroom-based positive psychology intervention workshop, embedded as an elective course into the regular curriculum of a medical school, significantly increased participating students’ sense of hope, life satisfaction, and subjective wellbeing, while reducing their levels of depressive and anxiety symptoms (Zhang et al., 2020). This demonstrates the effectiveness and feasibility of formalizing positive psychology content into courses. Furthermore, conducting short-term, thematic workshops is another efficient and flexible intervention model. For instance, a “Sleep Science and CBT-I Workshop” can address the common sleep problems among graduate students by providing scientific knowledge and practical skills based on cognitive behavioral therapy for insomnia (CBT-I) (Meaklim et al., 2023). Online workshops, due to their scalability and cost-effectiveness, are particularly suitable for dissemination among graduate student populations. An online sleep education seminar for graduate psychology students showed that the workshop effectively enhanced students’ sleep knowledge and their sense of self-efficacy in managing sleep disorders, with effect sizes reaching medium to large levels. Moreover, at the 12-month follow-up, 83% of the students applied the knowledge they gained in clinical practice (Meaklim et al., 2023). Similarly, a cognitive-behavioral therapy-based anxiety toolbox workshop for higher education students has also been proven effective in reducing students’ stress, emotional overload, and academic anxiety, while improving their time management skills and academic focus (Duraku et al., 2025). These thematic workshops are characterized by clear objectives, short duration, and strong practicality, enabling rapid improvement in students’ knowledge, self-efficacy, and willingness to apply knowledge practically in specific areas, serving as a powerful supplement to curriculum education.

### 5.2 Intervention pathways based on teacher-student relationships and peer support

In the graduate student mental health education system, supervisors and peers constitute two key sources of social support, and the intervention pathways built upon this foundation have profound impacts. The supervisor, as a pivotal figure in a graduate student’s academic career, plays a role that extends far beyond academic guidance. High-quality teacher-student intimacy can significantly enhance students’ interpersonal emotion regulation effects, which has a basis in neural synchrony (Johnson et al., 2025). Therefore, conducting specialized training for supervisors is crucial to enhance their ability to identify students’ mental states, engage in supportive communication, and act as “interpersonal emotion regulators.” Research indicates that virtual mental health literacy workshops designed for higher education staff can effectively improve their mental health knowledge and sense of self-efficacy in recommending mental health resources to students (Johnson et al., 2025). Such training helps supervisors become more acutely aware of students’ psychological distress and offer emotional support more effectively, thereby fostering a safe and supportive emotional environment in teacher-student interactions. Additionally, constructing a systematic peer support network is another core pathway. This can be achieved by establishing “senior student” mentorship programs, setting up graduate student psychological committee systems, or organizing regular peer support groups. Peer support, due to similarities in age, experience, and circumstances, is more likely to generate resonance and understanding. Online support groups demonstrate unique advantages in terms of resource accessibility and flexibility. For example, in resource-limited settings, mental health promotion workshops co-designed and led by young peer educators have successfully incorporated intergenerational learning, mobilized communities through participatory activities, and provided a feasible model for youth mental health promotion (Vostanis et al., 2026). Furthermore, during the pandemic, online support groups were proven to be a feasible, acceptable intervention form that could improve participants’ quality of life (Vostanis et al., 2026). This peer-based mutual aid model not only provides individuals with emotional support and practical advice but also reduces the stigma associated with psychological issues at the group level and encourages help-seeking behavior. Combining supervisor support with peer support can construct a multi-level, three-dimensional social support system for graduate students, jointly promoting their psychological resilience and healthy development through both formal and informal channels. Therefore, universities have the responsibility to go beyond general emotional care and systematically build a multi-level, multifaceted support network, ensuring that graduate students can access timely, effective, and tailored external resources when facing multiple challenges such as academic pressures, employment, and personal development, thereby significantly enhancing their overall psychological resilience and ultimately establishing a replicable and scalable positive support system that provides long-term and stable protective effects.

### 5.3 Integration and application of digital technology and online interventions

The rapid development of digital technology provides a powerful engine for innovation in graduate student mental health education. Integrating digital technology, particularly developing mobile health applications (mHealth Apps) or online platforms based on positive psychology theories, can overcome temporal and spatial limitations, offering students convenient, personalized, and readily accessible psychological resources. These applications can integrate modules such as mindfulness meditation exercises, gratitude journaling, identification of personal strengths and related challenges, and mood tracking, embedding mental health self-management into students’ daily lives. A case study targeting medical students demonstrated that participatory co-design workshops fostered strong student interest in designing mHealth applications for mental health promotion and proposed prototype ideas containing common themes such as personalization, data security, and scientific evaluation (Dederichs et al., 2022). This reflects students’ acceptance of and demand for digital mental health tools and suggests that educators can guide students to become active users and co-designers of technology through similar workshops. Beyond self-help tools, digital technology can also empower more complex teaching and collaboration scenarios. Utilizing online simulation training for interprofessional collaborative education is an innovative direction. For example, conducting psychopharmacology simulation training based on remote behavioral health among nursing and pharmacy graduate students can cultivate their respective professional skills while enhancing interprofessional collaboration abilities and adaptability to future complex work scenarios (Morley et al., 2025). A study evaluating an interprofessional education (IPE) workshop for nursing and pharmacy students found that the workshop significantly promoted positive working relationships and collaboration among students from different disciplines, facilitating effective sharing of knowledge and skills (Morley et al., 2025). Additionally, remote psychiatry simulation training has been used to train medical students in the safe use of telehealth technology and to practice handling high-risk situations such as suicide assessments, which are common in primary care and mental health telehealth clinics (Liew et al., 2022). These digital technology-based simulation training sessions create safe, controlled practice environments, greatly expanding the boundaries of traditional education and providing new pathways for cultivating graduate students’ comprehensive practical abilities and teamwork skills.

### 5.4 Building a campus culture and environmental support system

Campus culture and environment form the macro background and foundation for graduate students’ mental health education, and the creation of a supportive atmosphere is key to transitioning from individual intervention to systemic prevention. Deeply integrating mental health promotion into campus culture construction and institutional design aims to go beyond fragmented courses or activities to foster an atmosphere of full participation and continuous attention. Specific measures may include establishing a “Mental Health Awareness Month,” which centrally promotes mental health knowledge through a series of lectures, exhibitions, and experiential activities; creating relaxation and mindfulness spaces on campus to provide students with physical venues for stress relief and self-regulation; and promoting reforms in the academic evaluation system to encourage a more balanced and diverse view of development, so as to alleviate the pressure of a single outcome-oriented approach. These efforts aim to shape a campus culture that values psychological wellbeing, opposes stigma, and encourages help-seeking. For example, a campus mental health literacy program implemented in Barcelona, Spain, which provides long-term workshops for secondary school students, has shown that the program increases students’ mental health knowledge, improves help-seeking behavior, and reduces stigma (Casañas et al., 2020). Although targeting a younger demographic, its approach of creating a supportive environment through systematic campus projects offers valuable insights for graduate education as well. Simultaneously, the transformation of services at the university counseling center is crucial. Traditional counseling centers primarily offer individual crisis intervention and treatment, but from the perspectives of positive psychology and developmental approaches, there is a need to significantly strengthen developmental and preventive services. This includes implementing positive psychology training programs for a broad student base, thematic group counseling (such as stress management, interpersonal relationships, and career planning groups), and mental health education lectures. An evaluation of a trauma and mental health awareness education workshop for emergency service workers noted that workshops led by facilitators with personal experience, due to their high relevance and authenticity, created a positive learning experience and were an acceptable way to convey information about mental health, post-traumatic stress disorder (PTSD), and help-seeking (Fogarty et al., 2021). This indicates that group activities co-led by professionals and experienced individuals can effectively enhance participants’ awareness and skills. By expanding the function of the counseling center from “treatment” to “development” and “prevention,” and coordinating with the overall campus culture construction, a comprehensive, multi-level and long-term support environmental support system for graduate student mental health can be built, fundamentally promoting the psychological flourishing of the group.

## 6 Evaluation of the effectiveness, challenges, and optimization strategies of the practice pathway

### 6.1 Evaluation of intervention effectiveness based on the RE-AIM framework

The RE-AIM (Reach, Effectiveness, Adoption, Implementation, Maintenance) framework from knowledge translation research provides a scientific and multidimensional perspective for systematically evaluating the effectiveness of the graduate student mental health education practice pathway. This framework emphasizes a comprehensive consideration of the intervention’s reach, actual effectiveness, adoption level, implementation quality, and long-term sustainability from both individual and system/environmental levels (Cox-Martin et al., 2023). In the field of graduate student mental health education, applying this framework helps to move beyond the traditional limitation of focusing solely on whether an intervention is “effective,” and instead assesses the extent to which an intervention reaches the graduate student population in real, complex educational environments, how many institutions adopt and faithfully implement it, and whether its positive effects can be sustained long-term (Wu et al., 2022). For example, a RE-AIM evaluation of a psychosocial intervention for pain management in breast cancer survivors showed that the reporting rates for individual-level “Reach” and “Effectiveness” indicators were relatively high (65.2 and 62.3%, respectively). However, the reporting rates for system-level “Adoption,” “Implementation,” and “Maintenance” indicators were significantly lower (15.2, 46.8, and 7.7%, respectively), revealing the translational gap from effective research to sustainable practice (Cox-Martin et al., 2023). This suggests that when evaluating graduate student mental health programs, we should not only look at the scale of participating students (Reach) and symptom improvement post-intervention (Effectiveness), but also examine how many departments or supervisors are willing to adopt (Adoption) the program, whether the implementation process maintains fidelity (Implementation), and whether related practices and support systems can be sustained (Maintenance) after the program ends (Somerville et al., 2024). Regarding specific indicators for effectiveness evaluation, a diversified measurement system should be adopted. In addition to routinely monitoring the reduction of psychological distress symptoms such as anxiety and depression, greater emphasis should be placed on improving positive psychological indicators, such as psychological capital, subjective wellbeing, academic self-efficacy, and the quality of teacher-student relationships (Ver Hoeve et al., 2024). For example, an evaluation of a navigation intervention for community cancer patients found that the intervention not only significantly reduced the number of barriers faced by patients but also significantly improved their self-efficacy and satisfaction with communication from the medical team (Ver Hoeve et al., 2024). Similarly, a community intervention project targeting childhood obesity also reported improvements in the health-related quality of life of participating children (Briatico et al., 2023). These studies support the necessity of incorporating positive development indicators into the core evaluation scope of graduate student mental health intervention effectiveness, thereby more comprehensively reflecting the holistic value of educational interventions in promoting the healthy growth and potential development of graduate students.

### 6.2 Main challenges faced during implementation

Integrating a positive psychology-oriented mental health education pathway into the graduate student training system faces multiple practical challenges. The primary challenge is the scarcity of resources and professional expertise. Effective interventions, especially those based on evidence-based methods such as cognitive behavioral therapy (CBT), require practitioners to have appropriate professional competence (Meaklim et al., 2023). However, there is currently a severe shortage of faculty who are both systematically proficient in the theory and practice of positive psychology and have a deep understanding of the unique stressors and educational-cultural context of the graduate student population. An evaluation of an online sleep education workshop for psychology graduate students pointed out that although the workshop effectively enhanced students’ self-efficacy regarding cognitive behavioral therapy for insomnia (CBT-I), achieving clinical competence in CBT-I still requires more in-depth and longer-term professional practical training (Meaklim et al., 2023). This highlights the urgency and long-term nature of cultivating interdisciplinary professionals in the field of graduate student mental health education. Secondly, the challenge of systemic integration is particularly prominent. The core goal of the graduate training system is scientific research innovation and output. How mental health education can be organically and seamlessly embedded into this system, rather than being perceived as an “additional task” that increases the burden on teachers and students, is key to realizing its effectiveness. This requires intervention designs to integrate deeply with core aspects such as research training, supervisor guidance, and academic evaluation, exploring an integrated model of “psychological empowerment for research” to avoid low adoption rates and poor sustainability resulting from fragmentation (Walker et al., 2020). Finally, cultural differences and individual diversity cannot be ignored. Some core concepts and intervention techniques of positive psychology originate from Western cultural backgrounds. When introducing them to the Chinese graduate student population, careful assessment and adjustment for cultural adaptation are necessary (Sayarifard et al., 2024). For example, topics such as self-expression and the relationship between personal achievement and collective values may require localized interpretation. Simultaneously, there is significant diversity within the graduate student population. Students from different disciplines (e.g., STEM vs. humanities), different academic years (e.g., freshman adaptation period, mid-term assessment period, graduation and job-seeking period), different genders, and different family socioeconomic backgrounds have vastly different psychological needs, sources of stress, and coping resources (Parola, 2020). A study on Italian university graduates found that the economic uncertainty brought by the COVID-19 pandemic significantly exacerbated their anxiety and fear during the career planning process (Parola, 2020). This requires the mental health education pathway to possess sufficient flexibility and specificity, capable of providing differentiated and individualized support plans rather than a “one-size-fits-all” universal curriculum, in order to truly meet the real needs of diverse groups.

Building on this, to ensure that these interventions have a lasting impact, evaluations of mental health education interventions for graduate students must extend beyond immediate or short-term outcome measures. Long-term follow-up assessments must be prioritized to examine the sustainability of intervention effects and their profound influence on individual long-term development. Graduate education is a process that spans several years, during which stressors (such as graduation, employment, and career development) continuously evolve. The “attenuation effect” or “delayed effect” of short-term intervention outcomes needs to be revealed through long-term follow-up. Long-term longitudinal assessments aim to explore whether early mental health promotion interventions can provide graduate students with sustained psychological resources and buffers when confronted with more severe challenges later (such as writing a thesis, job interviews, or starting a career). For example, a literature review on veterinary students and graduates points out that balancing the prevention of mental illness with the promotion of psychological wellbeing is crucial. This implies that assessments need to focus on resources that can support long-term career wellbeing, such as professional competence, mentoring relationships, and positive help-seeking behavior (Bookbinder, 2025). Longitudinal studies can assess whether interventions positively affect graduate students’ graduation rates, employment quality, job satisfaction, and long-term risk of burnout. Furthermore, from a developmental perspective, the positive psychological qualities cultivated during graduate studies (such as psychological capital and a growth mindset) may have a cumulative effect on lifelong adaptation and wellbeing. Research indicates that graduate students with a growth mindset demonstrate greater resilience in research self-efficacy and anxiety levels regardless of the quality of student-teacher relationships, suggesting that early cultivation of a positive mindset has long-term protective effects (Li et al., 2025). Therefore, it is indispensable to establish an assessment framework incorporating multiple long-term follow-up time points (e.g., at graduation, 1 year post-graduation, and 3 years post-graduation) and to systematically collect data on various aspects such as subjective wellbeing, psychological capital, career adaptation, and physical health. This approach is vital for verifying the long-term value of mental health education interventions, optimizing resource allocation, and building an educational ecosystem that supports the lifelong development of graduate students.

### 6.3 Future optimization and development directions

To address the above challenges and promote the substantive progress of graduate mental health education, future efforts will require systematic optimization and innovation across multiple dimensions. The core objective is to transition educational practices from standardization toward precision and scientifically grounded approaches, thereby eliminating the “one-size-fits-all” model. A primary strategic priority is to foster robust interdisciplinary collaboration. Graduate student mental health is a complex issue involving knowledge of psychology, education, medicine (particularly mental health), management (organizational behavior), and even specific disciplinary fields. Therefore, it is essential to integrate multidisciplinary forces to develop, implement, and evaluate intervention programs (Fazel and Soneson, 2023). This collaboration is crucial for conducting an in-depth analysis of the unique psychosocial ecosystems in which different disciplines and groups operate. For example, veterinary students experience distinct forms of compassion fatigue, while nursing students are significantly affected by their perception of pandemic infection risks. These issues necessitate the collaborative design of tailored interventions by interdisciplinary teams comprising experts in clinical psychology, veterinary medicine, and nursing (Sosnicki and Reynolds, 2026; Graves et al., 2022). For instance, in the field of child and adolescent public mental health, researchers have called for a comprehensive research perspective encompassing interpersonal, community, and institutional systems (Fazel and Soneson, 2023). This interdisciplinary collaboration model is directly applicable to graduate student groups, bringing together clinical psychology experts, higher education researchers, student affairs administrators, and academic advisors to form an innovative collaborative alliance. This coalition lays the foundation for the development of highly targeted support programs.

Secondly, it is imperative to strengthen faculty training and professional support systems. This includes providing systematic training for graduate supervisors, counselors, and administrative staff covering the fundamentals of positive psychology, mental health first aid skills, and how to identify and support students in psychological distress (Greenberg et al., 2021). Empowering mentors and counselors to serve as pivotal agents in promoting graduate student mental health is crucial for building a supportive environment. Future training should prioritize identifying and responding to the diverse needs of student groups, such as the unique psychological challenges faced by international students, students experiencing delayed graduation, and part-time students (Wu et al., 2022). A study on workplace health promotion indicates that training middle managers and providing ongoing guidance can successfully drive the design and implementation of health promotion interventions (Greenberg et al., 2021). This underscores the fact that a well-trained teaching staff with competence in providing differentiated support is the critical human resource foundation for the implementation of categorized intervention plans and the realization of “warm” education.

Thirdly, emphasis should be placed on combining evidence-based practice with localized innovation. While actively introducing internationally proven effective intervention models (such as interventions based on Acceptance and Commitment Therapy), it is necessary to conduct empirical research based on the specific cultural, social, and educational contexts of Chinese graduate students to develop localized theoretical models and practical tools (Fung et al., 2021). Future research must develop and validate refined intervention programs based on the framework of pre-demand assessment, a classification system, and an academic year schedule. Examples include integrating embedded mental health education for clinical veterinary students (Sosnicki and Reynolds, 2026), constructing a dual pathway of resilience enhancement and social support for sports majors facing high employment pressure (Wang and Zhang, 2025), and enhancing hope and providing optimistic attribution training for graduating students. For example, a social media-based healthy lifestyle promotion intervention conducted among Chinese university students, evaluated using the RE-AIM framework, demonstrated good feasibility and effectiveness, providing an example for utilizing localized digital platforms for health interventions (Wang et al., 2021). This suggests that future evidence-based practice must rigorously incorporate the principles of “classification-based strategies, phased progression, and precise adaptation” to ensure that interventions are both empirically supported and tailored to the authentic contexts of diverse student populations.

Finally, efforts should be made to build a multi-level, whole-process support system. This means systematically integrating short-term thematic workshops, long-term developmental courses, environmental-level supportive policies (such as fostering a positive laboratory atmosphere and flexible academic systems), and professional counseling services to form a mental health support chain covering the entire process of graduate students, from enrollment adaptation, research challenges, and mid-term assessments to job hunting and graduation (Schlachter et al., 2024). The design of this support chain is designed to transition mental health education from remedial crisis intervention to proactive, full-cycle empowerment. It must address the distinct needs of students at various stages of their academic journey (e.g., higher risk near graduation (Wu et al., 2022)) and representing diverse identity backgrounds, thereby facilitating the precise allocation of support resources. For instance, a comprehensive intervention project for the mental health and re-employment of unemployed individuals attempted to provide personalized multi-level support by integrating three components: short-term psychotherapy, career coaching, and peer support (Schlachter et al., 2024). This systematic design of the support chain aims to shift mental health education from remedial crisis intervention to proactive whole-process empowerment, ultimately serving the comprehensive growth and sustainable development of the graduate student population. This systematic and categorized support framework is the institutional guarantee for ensuring the comprehensive growth and sustainable development of graduate students.

The optimization and innovation of future graduate mental health education must closely center on the core principle of “precision” (Differentiated), by deepening interdisciplinary cooperation to understand complex ecosystems, strengthening teacher training to enhance support capabilities, integrating evidence-based and context-specific research to develop tailored solutions, and ultimately constructing a categorized, whole-process support system to achieve a fundamental shift from “unified interventions” to “precision empowerment,” thereby effectively realizing high-quality, efficient, and human-centric educational goals.

## 7 Extension of interdisciplinary and specialized fields

### 7.1 Application in professional degree graduate students (e.g., medicine, veterinary medicine, psychology)

Graduate students in health-related fields such as medicine and veterinary medicine face extremely high levels of occupational stress and mental health risks. Research indicates that medical graduate students commonly experience significant psychological distress, including symptoms of depression, anxiety, and stress, which may intensify during clinical years (Atkinson, 2020). Veterinary students and graduates also face severe challenges, with mental health issues such as burnout, depression, and compassion fatigue being widely documented (Bookbinder, 2025). For these high-risk groups, the design of proactive and integrated interventions is crucial. For example, a “Compassion Fatigue Symposium” designed for veterinary clinical-year students has been shown to be an effective intervention. This proactive, integrated approach involves small group discussions led by clinical faculty, focusing on compassion fatigue, burnout, and self-care practices. Evaluations revealed that after participating in the symposium, students reported increased knowledge, confidence, and preparedness in addressing mental health challenges in veterinary medicine (Sosnicki and Reynolds, 2026). This provides preliminary evidence and practical guidance for integrating mental health education into clinical-year curricula. Furthermore, for medical graduate students, studies have found that adverse childhood experiences are associated with higher psychological distress. However, most students still report normal or high levels of psychological resilience, suggesting that educational interventions should simultaneously focus on trauma-informed care and resilience building (Sciolla et al., 2025). Therefore, designing structured, proactive psychological support programs for clinical-year students can effectively enhance their awareness and coping abilities regarding profession-specific mental health challenges.

The training of clinical psychology graduate students not only requires attention to their own mental health but also must strengthen their ability to serve culturally diverse clients. Cultural humility training is essential for enhancing their capacity to serve populations from different backgrounds and reducing inequalities in mental health services. Positive psychology concepts can help cultivate their self-efficacy and resilience in cross-cultural work. However, there are some gaps in the current training of clinical psychology doctoral students. For instance, a survey showed that although clinical psychology doctoral students are aware of the issue of “food insecurity,” which is closely related to sociocultural factors, the vast majority (over 80%) expressed a desire for training in related assessment and resource provision, while in reality, less than 10% of students had received such training during their graduate education (Browne et al., 2022). This highlights deficiencies in cultivating culturally responsive practice skills. Additionally, psychology graduate students also endure significant stress during training, including high workloads, financial distress, and challenges in relationships with supervisors, all of which may exacerbate anxiety and depressive symptoms (Hulac et al., 2025). Concepts emphasized by positive psychology, such as strengths, resilience, and self-efficacy, can serve as core components of training. By integrating cultural humility with positive psychology interventions—for example, fostering students’ psychological resilience in cross-cultural contexts, beliefs in self-efficacy, and the ability to utilize personal strengths—they can be better equipped to handle complex clinical work and provide a buffer for their own mental health in high-pressure training environments. This comprehensive training pathway aims to shape future practitioners who can provide effective, equitable mental health services while maintaining their own professional wellbeing.

### 7.2 Application in responding to specific crises (e.g., pandemics, employment pressure)

During sudden public health crises such as the COVID-19 pandemic, the mental health of graduate students has been significantly impacted, with positive psychological factors playing a crucial protective role. Multiple studies have confirmed that positive psychological resources such as hope, self-efficacy, and social support serve as important buffers in mitigating the negative effects of crises. For example, a study on medical students in the United States found that the COVID-19 pandemic led to a significant increase in perceived stress levels, with female gender and pre-existing mental health diagnoses being risk factors for elevated stress (Zhang et al., 2022). This suggests that educational institutions need to proactively strengthen students’ protective factors. Another survey of graduating health professional students revealed that the COVID-19 pandemic had widespread negative effects on their educational experience, leading to reduced educational opportunities, increased feelings of loneliness, feelings of defeat, and burnout (Clark et al., 2023). However, the study also found that higher resilience scores were associated with higher self-reported stress, fewer burnout symptoms, and better overall wellbeing (Clark et al., 2023). This indicates that even during crises, resilience, as a positive psychological quality, remains linked to better adaptation outcomes. In Lebanon, medical students and graduates facing multiple crises—the COVID-19 pandemic, economic collapse, and explosions—exhibited very high levels of psychological distress during e-learning (Bou Zerdan et al., 2023). These findings collectively emphasize that educational institutions should not merely react passively to crises but should actively and systematically integrate interventions. Such interventions aim to strengthen students’ sense of hope, cultivate their self-efficacy in facing challenges, and build robust peer and mentor social support networks, thereby effectively mitigating the long-term negative impact of crises on graduate students’ wellbeing.

In response to the widespread employment anxiety among graduate students, interventions based on positive psychology should focus on enhancing psychological resilience and the perception of social support. Research shows that psychological resilience plays a mediating role between academic stress and employment anxiety, and strengthening resilience is an effective way to alleviate employment anxiety. For medical graduate students, taking the postgraduate entrance examination is a critical milestone in their career status, with intense competition, and failure may lead to adverse mental health consequences. A study on Chinese medical students preparing for the postgraduate entrance examination found that during the exam period, 12.10% of students had mental health issues, with somatization being the most prominent symptom; 6 months after the exam, this proportion significantly decreased to 4.40% (Chen et al., 2022). This highlights the immediate impact of specific employment-related events (such as high-stakes exams) on mental health. Another qualitative study on Chinese medical graduate students revealed multiple sources of their psychological stress and pointed out that current mental health education often adopts passive forms such as manuals and lectures, lacking proactive, stratified interventions for students with different stress levels (Zhang et al., 2024). This provides direction for intervention design: a shift from passively providing resources to actively identifying problems and delivering feedback-based interventions is needed. Enhancing psychological resilience is central to coping with such stress. Resilience not only helps students buffer the direct transmission of academic stress to employment anxiety but also promotes the adoption of more positive coping strategies. Meanwhile, the perception of social support, including support from mentors, peers, and family, is a key external resource for alleviating employment uncertainty and economic concerns. Therefore, educational practices should integrate resilience training programs (such as cognitive-behavioral skills, mindfulness exercises) and structured social support systems (such as mentor groups, peer support networks), thereby systematically building graduate students’ psychological capital to face challenges in the employment market, reducing anxiety levels, and facilitating their smooth transition to the professional stage.

## 8 Conclusion

Positive psychology has paved an innovative path for graduate student mental health education, shifting from passively addressing problems to actively cultivating strengths. From an expert perspective, the core value of this paradigm shift lies in its systematic and developmental nature. It does not negate the necessity of traditional problem-oriented interventions but rather constructs a more comprehensive mental health spectrum—from preventing psychological distress to promoting psychological flourishing. Current theoretical frameworks, such as integrated psychological capital, the PERMA model, and self-determination theory, are attempting to establish a multi-level goal system, which requires us to precisely balance the emphasis on “preventing illness” and “promoting development” in practice. Solely emphasizing positive traits may overlook actual pressures and difficulties, while focusing only on problems may limit individuals’ growth potential. Therefore, effective educational practices must be a dynamic balancing process, cultivating qualities such as optimism and resilience while also providing practical support tools for coping with real challenges like academic setbacks and interpersonal pressures.

Diverse practical approaches, ranging from curriculum design and optimizing teacher-student relationships to the application of digital tools, demonstrate the richness of intervention methods. However, the key to their success lies in achieving deep integration rather than simple addition. For example, online tools improve accessibility but require vigilance against the risk of weakening deep interpersonal connections. Fostering a supportive culture is fundamental, but without specific, structured programs to carry it out, it can easily become superficial. This requires educators to possess interdisciplinary perspectives, organically integrating the principles of positive psychology into the entire process of graduate student development, rather than treating it as an independent add-on module.

Looking ahead, the development of this field urgently needs to strengthen its evidence-based foundation and long-term mechanism construction. Adopting scientific evaluation frameworks such as RE-AIM is crucial, as it helps us move beyond “whether it is effective” to a more nuanced analysis of “under what conditions, for whom, and in what ways it is effective,” thereby balancing potentially contradictory findings across different studies. The professional capacity building of faculty is both a bottleneck and a breakthrough point. Only when mentors and administrators themselves understand and practice the concept of positive development can interventions have a profound impact. Ultimately, the significance of positive psychology-oriented mental health education extends beyond promoting individual physical and mental health and academic success. It is a strategic investment in enhancing the psychological resilience of high-level national talent and stimulating their innovative vitality and enduring motivation. By constructing a comprehensive support system spanning from admission adjustment to career development, we are not only cultivating healthier graduate students but also shaping future researchers and societal pillars who can tackle complex challenges and possess thriving vitality.

## Data Availability

The original contributions presented in the study are included in the article/supplementary material, further inquiries can be directed to the corresponding author.
